# A comprehensive analysis of allele-specific expression and transcriptomic profiling in pig limbic and endocrine tissues

**DOI:** 10.3389/fnmol.2025.1616363

**Published:** 2025-09-03

**Authors:** Muhammad Arsalan Iqbal, Frieder Hadlich, Henry Reyer, Michael Oster, Nares Trakooljul, Klaus Wimmers, Siriluck Ponsuksili

**Affiliations:** ^1^Integrative Genomics, Research Institute for Farm Animal Biology (FBN), Dummerstorf, Germany; ^2^Faculty of Agricultural and Environmental Sciences, University Rostock, Rostock, Germany

**Keywords:** pig, ASE, brain, endocrine, hypothalamic-pituitary-adrenal (HPA), limbic, diencephalon

## Abstract

**Introduction:**

Stress involves complex interactions between the brain and endocrine systems, but the gene-level processes and genetic factors mediating these responses remain unclear. This study investigates gene expression patterns and allele-specific expression (ASE) in key limbic, diencephalon and endocrine tissues to better understand stress adaptation at the molecular level.

**Methods:**

We performed RNA sequencing on 48 samples from six distinct tissues: amygdala, hippocampus, thalamus, hypothalamus, pituitary gland, and adrenal gland. These tissues were categorized into three functionally and anatomically distinct groups: limbic (amygdala, hippocampus), diencephalon (thalamus, hypothalamus), and endocrine (pituitary, adrenal). Differential expression analyses were conducted both between individual tissues and across these tissue groups. Weighted Gene Co-expression Network Analysis (WGCNA) was applied exclusively at the tissue group level to identify group-specific gene networks. Allele-specific expression (ASE) was analyzed at the individual tissue level to capture cis-regulatory variation with high resolution.

**Results:**

Thirty-three candidate genes were differentially expressed across all tissues, indicating a core set involved in stress responses. Weighted Gene Co-expression Network Analysis revealed limbic and diencephalon modules enriched in neural signaling pathways such as neuroactive ligand-receptor interaction and synaptic functions, while endocrine modules were enriched for hormone biosynthesis and secretion, including thyroid and growth hormone pathways. Over 1,000 genes per tissue showed ASE, with 37 genes consistently colocalized. Ten of these displayed differences in allelic ratios, with seven (*PINK1, TTLL1, SLA-DRB1, HEBP1, ANKRD10, LCMT1*, and *SDF2*) identified as eQTLs in pig brain tissue within the FarmGTEx database.

**Conclusion:**

The findings reveal significant genetic regulation differences between brain and endocrine tissues, emphasizing the complexity of stress adaptation. By identifying key genes and pathways, this study provides insights that could aid in enhancing animal welfare and productivity through targeted modulation of stress-related molecular pathways.

## 1 Introduction

Understanding pig stress responses is vital for improving animal welfare and productivity in farm settings. The cognitive and regulatory processes in a pig's brain underlie a complex stress response system that enables the animal to assess and cope with environmental challenges. Stressors, which are external factors that compromise the animal's physical or psychological condition, activate this system. These may include overcrowding, abrupt weaning, transportation, loud or unpredictable noise, rough handling, social isolation, and a lack of environmental enrichment (Lisowski et al., 2011; Perdomo-Sabogal et al., 2022; Papatsiros et al., 2024; Ajay et al., 2025). The process of controlling these stress responses is regulated by the coordination of the limbic system, including structures such as the amygdala, hippocampus, thalamus, hypothalamus, and the hypothalamus–pituitary–adrenal axis (HPA axis) (Mormède et al., 2007; Mormède, 2008). As mammals, pigs share these limbic structures with other species, including humans, reflecting an evolutionarily conserved mechanism underlying emotional and physiological responses to stress (Kanitz et al., 2019). This homology strengthens the translational value of pig models for studying stress-related neurobiological processes (Lind et al., 2007; Gimsa et al., 2018). Several studies have previously reported that the limbic system and endocrine glands are central to the health and optimal performance of farm animals, as they regulate stress responses, growth, and overall physiological balance (Manteuffel, 2002; Smith and Vale, 2006; MohanKumar et al., 2012; Gley et al., 2021).

Advancements in high-throughput sequencing have greatly enhanced transcriptomic analysis, providing deeper insights into gene expression dynamics and driving interest in allele-specific expression (ASE) research. Pigs, as important farm animals with significant economic and biomedical relevance, present a need to better understand the genetic regulation underlying complex traits such as growth, reproduction, and disease resistance (Mote and Rothschild, 2020). Traditional genetic studies alone often fall short in explaining this regulatory complexity, prompting a demand for tools that offer finer-resolution insights into gene expression control (Connally et al., 2022). The use of RNA sequencing in livestock research has expanded beyond traditional gene expression profiling, enabling cost-effective variant detection. It offers an alternative to whole-genome sequencing while overcoming the limitations of exome sequencing technologies (Han et al., 2015; Jehl et al., 2021). By analyzing transcriptomic data, variants within expressed regions can be identified, allowing for the investigation of cis-regulated genes through ASE analysis, a method that has become widely used since the introduction of RNA-seq technology (Montgomery et al., 2010; Pickrell et al., 2010; Battle et al., 2014; Deelen et al., 2015; GTEx Consortium, 2020). The use of RNA sequencing enables the differentiation of transcripts derived from an individual's two haplotypes using heterozygous markers, making ASE an effective tool for studying cis-regulatory variation. The strength of this approach lies in its ability to detect regulatory differences directly from transcribed regions, offering high-resolution, gene-specific insights in a cost-effective manner even with modest sample sizes. This enables the identification of functional variants with greater precision than broader eQTL studies, supporting more informed breeding strategies and improved trait selection (Schliekelman, 2008; Chamberlain et al., 2015; Pirinen et al., 2015).

Prior studies have well-documented that ASE detection from RNA sequencing is reliable when there is a heterozygous site in the gene's cis-regulatory region (Jehl et al., 2021; Iqbal et al., 2023). Studies in mice and humans increasingly show that variations in regulatory mechanisms, affect gene expression levels, detected as an allele-specific expression or allelic imbalance (Serre et al., 2008). For instance, a study found that over 80% of mouse genes exhibit cis-regulatory variation (Crowley et al., 2015). A comprehensive human study by the GTEx Consortium used transcriptomic data from various tissues including 11 brain regions to investigate gene expression and ASE across tissues (GTEx Consortium et al., 2015). Similarly, the Farm Animal GTEx (FarmGTEx) project has created an atlas of regulatory variants for domestic animal species. Notably, PigGTEx resources are freely accessible at http://piggtex.farmgtex.org (Teng et al., 2024).

ASE detection methods have evolved, from analyzing individual samples using tools like QuASAR (Harvey et al., 2015), to evaluating ASE across single nucleotide polymorphisms (SNPs) within a gene with methods like MBASED (Mayba et al., 2014) and GeneiASE (Edsgärd et al., 2016). Recently, ASEP (Allele-Specific Expression Analysis in a Population) has enabled ASE detection across multiple individuals, utilizing a generalized linear mixed-effects model that accounts for correlations of SNPs within the same gene (Fan et al., 2020).

In this study, we used RNA sequencing to analyze transcriptomic profiles and ASE across six tissues from the brain and endocrine system: amygdala (Amy), hippocampus (Hip), thalamus (Tal), hypothalamus (HT), pituitary gland (PG), and adrenal gland (AG). A total of 48 samples (8 per tissue) from the same animals minimized genetic variability and environmental noise, enhancing the robustness of our analysis. Differential expression analysis was performed both between individual tissues and between neuroanatomically and physiologically defined tissue groups (limbic, diencephalon, and endocrine). WGCNA was conducted at the tissue group, enabling broader detection of co-expression patterns and functional enrichments. ASE analysis at the individual tissue level revealed over 1,000 genes with allele-specific expression per tissue, including 37 shared across tissues. These shared ASE genes were further assessed for tissue-specific allelic ratios and functional relevance.

Through integrated analysis of gene expression, differential expression, WGCNA, ASE, and functional enrichment, our study uncovers distinct molecular signatures related to stress response, growth, and homeostasis across limbic, diencephalon, and endocrine tissue groups in livestock. We identified gene networks and pathways reflecting their specialized roles, such as neuronal signaling in limbic and diencephalon tissues and hormone biosynthesis in endocrine tissues. These insights offer valuable foundations for improving breeding and productivity while highlighting strategies to reduce environmental stress, enhance living conditions, and promote animal welfare and sustainable farming.

## 2 Materials and methods

### 2.1 Tissue collection

The study included 8 female German Landrace pigs, with an average age of 170 ± 14 days and weight of 105 ± 8 kg. The brain tissue and endocrine glands, including the Amy, Hip, Tal, HT, PG, and AG were swiftly removed, flash-frozen in liquid nitrogen, and preserved at −80 °C for subsequent analyses. The pig brain atlas was used to help with the dissection of different brain regions (Félix et al., 1999).

### 2.2 RNA extraction, library preparation, and data pre-processing

For consistency of the results, all tissue samples were taken from the left side of the organ, including the Amy, Hip, Hip, and Tal, while an entire organ of the PG and AG was used. Firstly, the tissue sample was finely ground into powder in liquid nitrogen. Total RNA was purified using the RNeasy Mini Kit (Qiagen, Germany) and DNase I treatment to remove trace genomic DNA contamination. A NanoDrop ND-1000 spectrophotometer (Peqlab) and a Bioanalyzer 2100 (Agilent Technologies) were used to determine the concentration and quality of RNA, respectively. One microgram (μg) of total RNA (RIN > 8) was used to generate the library using an Illumina Stranded mRNA Prep, Ligation with the kit with 11 cycles of PCR amplification as directed by the manufacturer's recommendation (Illumina, USA). The adaptor-ligated DNA libraries uniquely tagged with Ilumina Unique Dual (UD) index were quality checked, normalized, pooled, and sequenced for 2 × 101 cycles paired-end reads at 750 pM final concentration on the NextSeq 2000 system using a P3 flowcell. Library preparation and sequencing have been carried out at the sequencing facility of the Research Institute for Farm Animal Biology (FBN), Dummerstorf, Germany. Raw sequencing reads (fastq) were generated using dragen bcl convert v3.10.11 and quality-checked using FastQC version 0.11.9 (Andrews, 2010).

Data preprocessing was performed using Trim Galore (version 0.6.10.). Low-quality reads (a mean Q-score < 30) and short reads (< 20 bp) as well as adapter-like sequences at the 3′-end of sequence reads were removed (Krueger, 2015). Afterward, high-quality paired-end reads were then aligned to the Sscrofa11.1 reference genome (ENSEMBL release 105) using nf-core rnaseq pipeline (version 3.4) with STAR aligner (version 2.7.8a) and Salmon (version 1.10.2) (Dobin et al., 2013; Patro et al., 2017; Ewels et al., 2020). The RNA sequencing data obtained were deposited in the ArrayExpress database under the provided accession: E-MTAB-14452.

### 2.3 Gene expression profiling and downstream analysis

After pre-processing the count data, it was transformed into a variance-stabilized format. Principal Component Analysis (PCA) was subsequently performed on a variance-stabilized expression matrix using the *prcomp()* function in base R, which applies singular value decomposition (SVD) to identify orthogonal components that capture maximum variance. The first two principal components were used to visualize sample clustering and variance structure across tissues. PCA was performed both within individual tissues and across grouped tissue categories: limbic (Amy and Hip), diencephalon (Tal and HT), and endocrine (PG and AG), enabling broader detection of co-expression patterns and functional enrichments. Pairwise differential expression analysis was conducted using the DESeq2 package (version 1.42.0) in the R programming environment (Love et al., 2014). Two categories of comparisons were performed: (1) individual tissue comparisons (among each of the six distinct tissue types), and (2) tissue group comparisons (between biologically or functionally related tissue). The differential expression model was defined as:


(1) $$\begin{array}{l} Y=\;\beta 0+\;\beta 1\cdot Tissue\;or\;Tissues\;Group+E \end{array}$$

Where Y is the gene expression level, β0 is the intercept, β1 represents the effect of each tissue type or tissue group, and ε is the error term.

Differential expression analysis was performed using the Wald test within DESeq2. For individual tissue comparisons, differentially expressed genes (DEGs) were identified based on an adjusted *p*-value (FDR) < 0.05. For tissue group comparisons, a more stringent threshold was applied: genes were considered differentially expressed if they met both FDR < 0.05 and an absolute log2 fold change (|log_2_FC|) ≥ 2. In total, 15 pairwise comparisons were conducted between individual tissues, resulting in 15,516 unique DEGs. These genes were used to explore gene expression overlaps and similarities across tissues. Hierarchical clustering of these DEGs was performed using the *heatmap.2()* function from the gplots package (version 3.1.3) (Warnes et al., 2016) to identify co-expression patterns across tissues. These clustered genes underwent KEGG pathway enrichment analysis using the ClueGO (version 2.5.10) and CluePedia (version 1.5.10) plugins in Cytoscape (version 3.10.2) (Shannon et al., 2003; Bindea et al., 2009, 2013) with pathway significance determined by a hypergeometric test followed by Benjamini–Hochberg correction (*p* < 0.05).

To visualize overlaps between the 15 comparison groups, DEGs from each were analyzed with the EVenn web tool (Chen et al., 2021). Flower plots were used to depict shared genes, which were further analyzed for expression patterns across tissues. Log fold change (logFC) for each gene was calculated relative to the average expression across all tissues, and this was visualized in a heatmap generated by the pheatmap package (version 1.02.12) within the R programming environment (Kolde and Kolde, 2015). The differentially expressed genes from each pairwise tissue group comparison were visualized using volcano plots generated with the ggplot2 package (version 3.5.1) (Wickham et al., 2016).

### 2.4 Functional annotation of DEGs from tissue group pairwise comparisons

Differentially expressed genes (DEGs) between brain tissue groups (limbic, diencephalon, and endocrine) were analyzed for functional enrichment with clusterProfiler package (version 4.12.6) within the R programming environment (Yu et al., 2012). The DEG sets for each pairwise comparison were divided into upregulated and downregulated genes based on log2 fold change. The KEGG pathway and Gene Ontology biological Process (GO BP) enrichment analyses were performed using the enrichKEGG() and enrichGO() functions, respectively, within the clusterProfiler package, using the pig genome. Subsequently, results were filtered to include only those with an adjusted *p*-value (Benjamini-Hochberg correction) < 0.05. For each significantly enriched pathway or GO term, enrichment scores were calculated as the negative log10 of the adjusted *p*-value (*p*.adj). These scores were used to construct tissue-specific composite enrichment scores for each term as follows:

Let L vs. D, L vs. E, and D vs. E represent the enrichment scores for the pairwise comparisons between Limbic vs. Diencephalon, Limbic vs. Endocrine, and Diencephalon vs. Endocrine, respectively. Composite scores for each tissue group were calculated as follows: Limbic score = (L *vs*. D) + (L vs. E), Diencephalon score = – (L vs. D) + (D *vs*. E), and Endocrine score = – (L vs. E) – (D vs. E). These composite scores capture the direction and magnitude of KEGG pathway and biological process enrichment for each tissue group relative to the others. A positive enrichment score in a pairwise comparison indicates greater enrichment in the first tissue listed. Heatmaps of the composite scores were generated using the pheatmap package (version 1.0.13) in R.

### 2.5 Gene co-expression analysis with WGCNA

An expression matrix of differentially expressed genes derived from the three pairwise tissue group comparisons was constructed. Co-expression network analysis was then performed using the Weighted Gene Co-expression Network Analysis (WGCNA) package (version 1.72-1) in the R programming environment (Langfelder and Horvath, 2008). To assess scale-free topology, the soft-thresholding power (β) was selected using the *pickSoftThreshold()* function. Network construction and module identification were carried out using the *blockwiseModules()* function, with the following parameters: power = 20, network type = signed, minimum module size = 100, and maximum block size = 1,500.

Module eigengenes (MEs), representing the first principal component of each module's gene expression profile, were computed using the *moduleEigengenes()* function. Pearson correlations between MEs and tissue group categories (limbic, diencephalon, and endocrine) were calculated using the *cor()* function. The significance of each correlation was assessed using Student's *t*-distribution through the *corPvalueStudent()* function. Modules with a correlation *p*-value < 0.05 were considered tissue group-specific. Genes from these modules were analyzed for KEGG pathway enrichment using the clusterProfiler package (version 4.12.6) within R, with the pig (*Sus scrofa*) database. Pathways with a false discovery rate (FDR) adjusted *p*-value < 0.05 were considered significantly enriched.

### 2.6 RNA-seq-based variant identification with GATK

Standard preprocessing of sequenced reads was performed according to Genome Analysis Toolkit (GATK, version 4.2.0.0) best practices guidelines, ensuring robust variant detection and genotype calling based on RNA sequencing data (Brouard et al., 2019; Jehl et al., 2021). The alignment of sequencing reads to the *Sus scrofa* reference genome assembly (Sscrofa11.1, ENSEMBL release 106) was performed using the STAR aligner (version 2.7.8a) with a 2-pass mode configuration (Dobin et al., 2013). Quality filtering was conducted on aligned BAM files to retain reads meeting the predefined quality criteria, and weighted analysis to account for selection and population structure (WASP) filtering was applied to select the high-quality alignment by samtools (version 1.12), as well as duplicate reads were identified and removed using “GATK MarkDuplicate” to obtain refined BAM files with unique and high-quality alignments. After alignment and quality filtering, the reads with “NS” in their cigar strings were split using “SplitNCigarReads,” which enabled thorough analysis of the splicing events, and base recalibration was performed using known variants from Ensembl v102's dbSNP (Hunt et al., 2018).

For variant identification, the “GATK's Haplotypecaller tool” was employed to detect single nucleotide polymorphisms (SNPs) and insertion/deletions (Indels) via the localized *de novo* assembly within active regions by applying a minimum-confidence threshold of 20, with the exclusion of soft-clipped bases (Van der Auwera et al., 2013). Subsequently, variant filtration was performed with “GATK's VariantFiltration” tool, applying defined parameters, including cluster window size of 35, cluster size of 3, and filter expression for two specific annotation FS (Fisher Strand) > 30 and QD (Quality by Depth) < 2 for detection and exclusion of variants with strand bias and/or low quality.

### 2.7 Analysis of allele-specific expression

For ASE from the SNP variant, an additional iteration of the GATK best practice pipeline was implemented. SNPs identified in the initial round (aforementioned in Section 1.5), underwent N-masking within the reference genome using bedtools (version v2.27.1), a crucial step to enable unbiased STAR alignment by the inclusion of the masked SNPs in the mapping process. Alongside the quality control procedures performed earlier, SNPs subjected to ASE analysis underwent filtration based on their read coverage. Only biallelic loci with heterozygosity with a minimum of 50 reads in total, at least 10 reads per allele, and with each allele contributing no < 1% to the total read count as well as SNPs on sex chromosomes and unmapped regions were removed from the further downstream analysis.

Additionally, tissue-specific gene-wise ASE analysis was performed in the Amy, Hip, Tal, HT, PG, and AG using the ASEP (Allele-Specific Expression Analysis in a Population, version 0.1.0) package within the R programming environment (Fan et al., 2020). The “*ASE_detection()*” functions were applied to identify gene-level ASE effects with statistical significance (*p*-value < 0.05) within each tissue. The analyses were performed using unphased, adaptive configuration with a resampling rate of 1e4. The genes exhibiting ASE from six different tissues (Amy, Hip, Tal, HT, PG, and AG) were analyzed with the EVenn web tool (Flower plot) to visualize the overlapping genes across all tissues. Furthermore, differential ASE detection was applied to pairwise tissue comparisons, and resulting shared genes are visualized in a heatmap using the gplots package (version 3.1.3) within the R environment.

Furthermore, to evaluate the overlap between ASE genes from the Amy, Hip, Tal, and AG (*p* < 0.05) and pig brain eQTLs, we accessed cis-eQTL data from the PigGTEx portal within the FarmGTEx database [Farm Animal Genotype-Tissue Expression database (Teng et al., 2024)]. We examined the PigGTEx_v0.permutations_eQTL file, specifically targeting the Brain.cis_qtl_fdr0.05 dataset, which was filtered for FDR < 0.05. Using a gene-matching strategy, we identified overlapping genes between the ASE genes in these tissues and the brain-specific eQTLs. Similarly, we sourced eQTL data for the hypothalamus and pituitary gland (FDR < 0.05) from the PigGTEx portal and confirmed the overlap between these eQTLs and the ASE genes identified in our study for HT and PG. The shared between eQTLs and the ASE genes identified from these tissues (Amy, Hip, Tal, HT, PG, and AG) were visualized using a circos plot, created with the circlize package (version 0.4.16) in the R environment (Gu et al., 2024).

### 2.8 Variance analysis of allelic ratios in ASE genes

The allelic ratio (AR) for each gene within tissue samples was calculated by dividing the sum of reference allele counts by the sum of total allele counts across all SNPs in the gene.


(2) $$\begin{array}{l} AR=\frac{\sum \mathrm{Ref}\left(a\right)}{\sum Total\left(b\right)} \end{array}$$

Where ∑Ref (a) is the sum of reference allele counts for all SNPs in the gene, and ∑Total (b) is the sum of total allele counts for all SNPs in the gene.

Genes with ASE overlapping across six tissues were selected, and their mean allelic ratio for each tissue was calculated to visualize the allele expression profile using a heatmap created with the pheatmap package (version 1.0.12) within the R environment. The “*aov()*” function in the R environment was applied to perform an analysis of variance (ANOVA) on the gene with ASE overlapped across six tissues (Amy, Hip, Tal, HT, PG, and AG) to evaluate the impact of tissue type on allelic ratio variation of each gene (St and Wold, 1989). Additionally, the mean allelic ratio and its standard deviation (SD) for shared genes were calculated across the six tissue types and used for pairwise comparisons through two-way ANOVA with Tukey's multiple comparison tests, conducted in the GraphPad prism. The significant differences in pairwise comparison and/or overall tissue type impact on gene allelic ratio were determined with a threshold of *p* < 0.05. The bar plots were generated using GraphPad prism, highlighting the observed differences in mean allelic ratio across different tissue types (Wickham et al., 2016).

## 3 Results

A comprehensive analysis was performed on transcriptome data collected from the pig's limbic system organs: Amy, Hip, Tal, HT, and the endocrine glands: PG and AG. A total of 1,128.8 million raw reads were generated from the mRNA sequencing of 48 libraries. These libraries were equally divided among six tissues Amy, Hip, Tal, HT, PG, and AG with 8 samples per tissue. An average of 23.5 million reads per sample was aligned to the Sscrofa11.1 reference genome (ENSEMBL release 105). Of these, an average of 21.1 million reads per sample were aligned, with 89.7% of the reads mapped in Supplementary Table S1. The resulting 30,635 genes (transcripts) that passed quality control were used for further analysis. Furthermore, two approaches were applied based on RNA-seq data: (1) gene expression analysis, which was used to identify expression changes between various tissues as well as among biologically or functionally related tissue groups, and (2) variant discovery analysis, which aimed to determine ASE within the tissues. Variance-stabilized expression values of a total of 30,635 genes underwent pairwise differential gene expression analysis across different tissues. Subsequently, a total of 95,033 commonly identified SNPs across all tissues were then analyzed to assess the ASE within the tissues.

### 3.1 Clustering of tissues based on transcriptomic data

**Figure 1:**
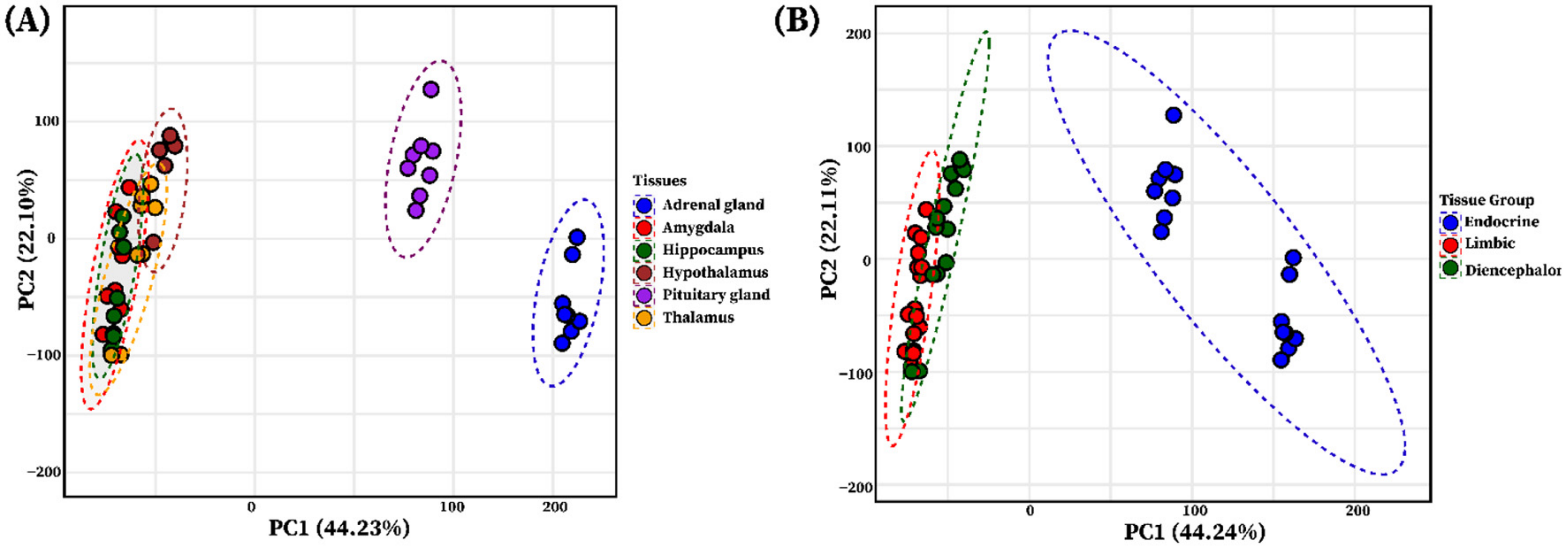
Principal component analysis (PCA) of individual tissues and functional tissue groups. **(A)** The PCA plot illustrates the clustering of tissue using the variance stabilized gene expression data, with each limbic system tissue and endocrine system glands represented by a distinct color circle: blue (adrenal gland), red (amygdala), green (hippocampus), brown (hypothalamus), purple (pituitary gland), and yellow (thalamus). **(B)** PCA of grouped tissues based on shared functional roles: Limbic system (amygdala and hippocampus, red), Diencephalon (thalamus and hypothalamus, dark green), and Endocrine (adrenal gland and pituitary gland, blue), showing group-level clustering.

PCA based on variance-stabilized counts from six tissues (Amy, Hip, Tal, HT, PG, AG) revealed distinct clustering patterns. The PCA indicated that PC1 and PC2 represent 44.23% and 22.10% of the total variance, respectively (Figure 1A). The analysis revealed distinct clustering patterns across tissues, with limbic system organs (Amy, Hip, Tal, HT) tightly grouped, and endocrine tissues (PG and AG) forming a separate cluster. To explore higher-level transcriptional organization, tissues were categorized into three functional groups: limbic (Amy and Hip), diencephalon (Tal and HT), and endocrine (PG and AG). PCA performed on these groups showed that the limbic and diencephalon groups were relatively close but remained separated, while both were distinctly clustered from the endocrine group (Figure 1B).

### 3.2 Expression clustering and pathway enrichment of DEGs across tissues

Fifteen pairwise comparison groups were analyzed, including Amy vs. Hip, Amy vs. HT, Amy vs. PG, Amy vs. AG, Amy vs. Tal, Hip vs. HT, Hip vs. PG, Hip vs. AG, Hip vs. Tal, HT vs. PG, HT vs. AG, HT vs. Tal, PG vs. AG, PG vs. Tal, and AG vs. Tal. The number of genes significantly differentially expressed at an FDR < 0.05, or at FDR < 0.05 with a log_2_ fold change (|log_2_FC|) ≥ 2, for each comparison group is shown in Table 1. Finally, the genes were aggregated, resulting in a total of 15,516 differentially expressed genes, covering all 15 of the comparison groups, outlined in Supplementary Table S2.

**Table 1:** The number of differentially expressed genes between different tissues with FDR < 0.05 and |log_2_FC| ≥ 2.

**Pairwise comparison groups**	**DEGs (FDR < 0.05)**	**DEG (FDR < 0.05 and |log_2_FC| ≥2)**
Amy vs. Hip	1,803	139
Amy vs. HT	10,520	954
Amy vs. PG	10,125	2,820
Amy vs. AG	10,393	3,915
Amy vs. Tal	3,792	754
Hip vs. HT	11,548	1,323
Hip vs. PG	10,615	3,008
Hip vs. AG	10,308	3,809
Hip vs. Tal	3,223	663
HT vs. PG	10,273	2,462
HT vs. AG	12,119	4,349
HT vs. Tal	8,991	700
PG vs. AG	10,320	2,900
PG vs. Tal	9,177	2,883
AG vs. Tal	9,349	3,666

Amy, Amygdala; Hip, Hippocampus; Tal, Thalamus; HT, Hypothalamus; PG, Pituitary Gland; AG, Adrenal Gland.

**Figure 2:**
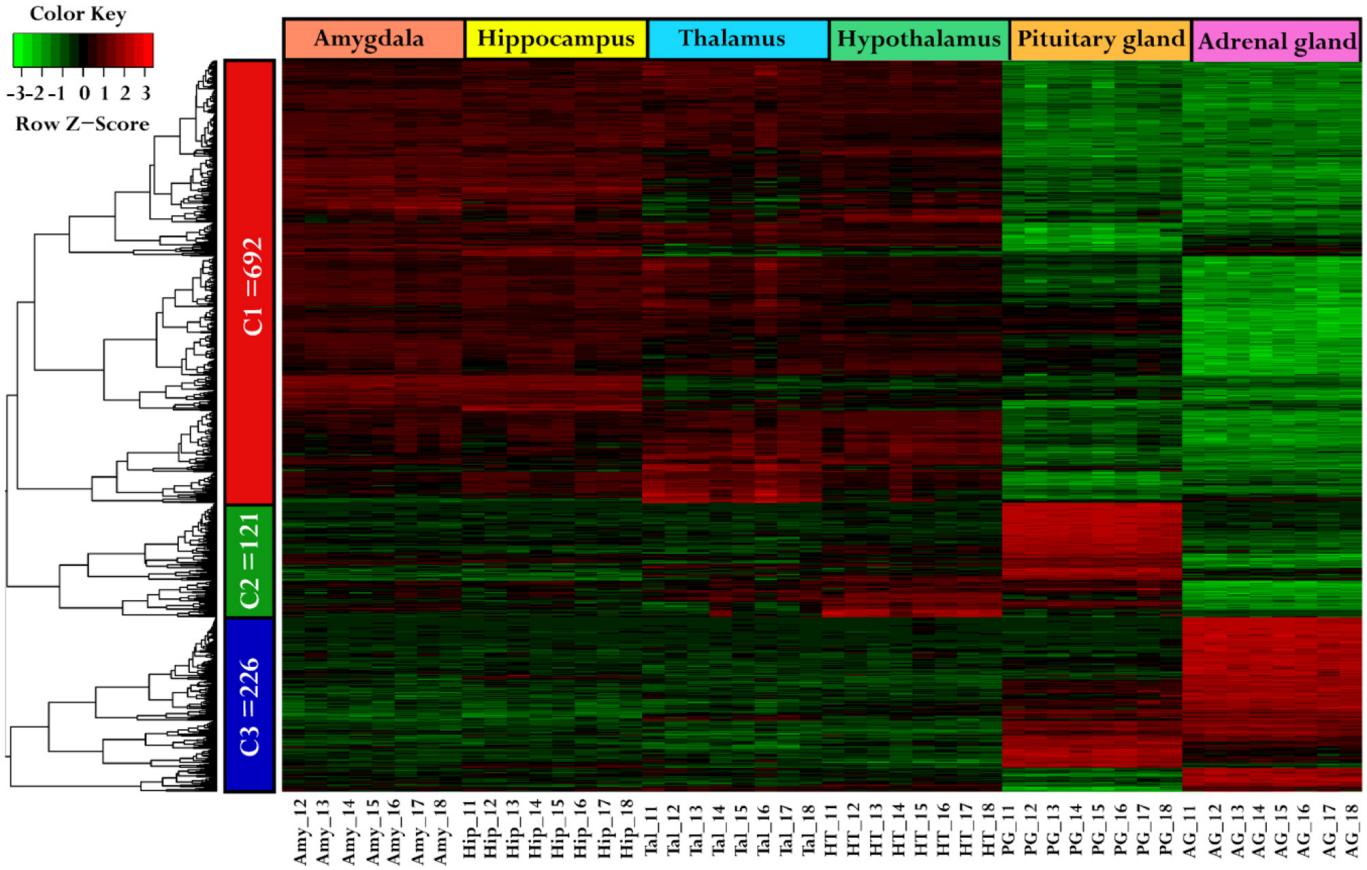
Comparative gene expression patterns among tissues. The heatmap was generated using hierarchical clustering, illustrating the gene expression variation across different tissues. The genes were categorized into three clusters: C1 (red), C2 (green), and C3 (blue). The tissues were represented by specific colors: the amygdala (darksalmon), hippocampus (yellow), thalamus (cyan), hypothalamus (green), pituitary gland (orange), and adrenal gland (darkpink). In the color key, red color indicates upregulation, green indicates downregulation, and black signifies no change in expression.

Using a hierarchical clustering heatmap to identify expression patterns across six different tissues, a total of 1,039 genes were grouped into three clusters based on their expression profiles, with a cutoff criteria of log2FC ≥ 2 and FDR < 0.05. Cluster one (C1) includes 692 genes, cluster two (C2) includes 121 genes, and cluster three (C3) includes 226 genes, as shown in Figure 2, and a list of cluster genes was provided in Supplementary Table S3. Moreover, the analysis suggests that the genes in C1 demonstrated similar expression patterns (higher expression) in the Amy, Hip, Tal, and HT as compared to the PG and AG. The genes in C2 show higher expression only in the pituitary gland compared to other tissues, while genes in C3 show upregulation in both the pituitary gland and adrenal gland compared to other tissues.

**Figure 3:**
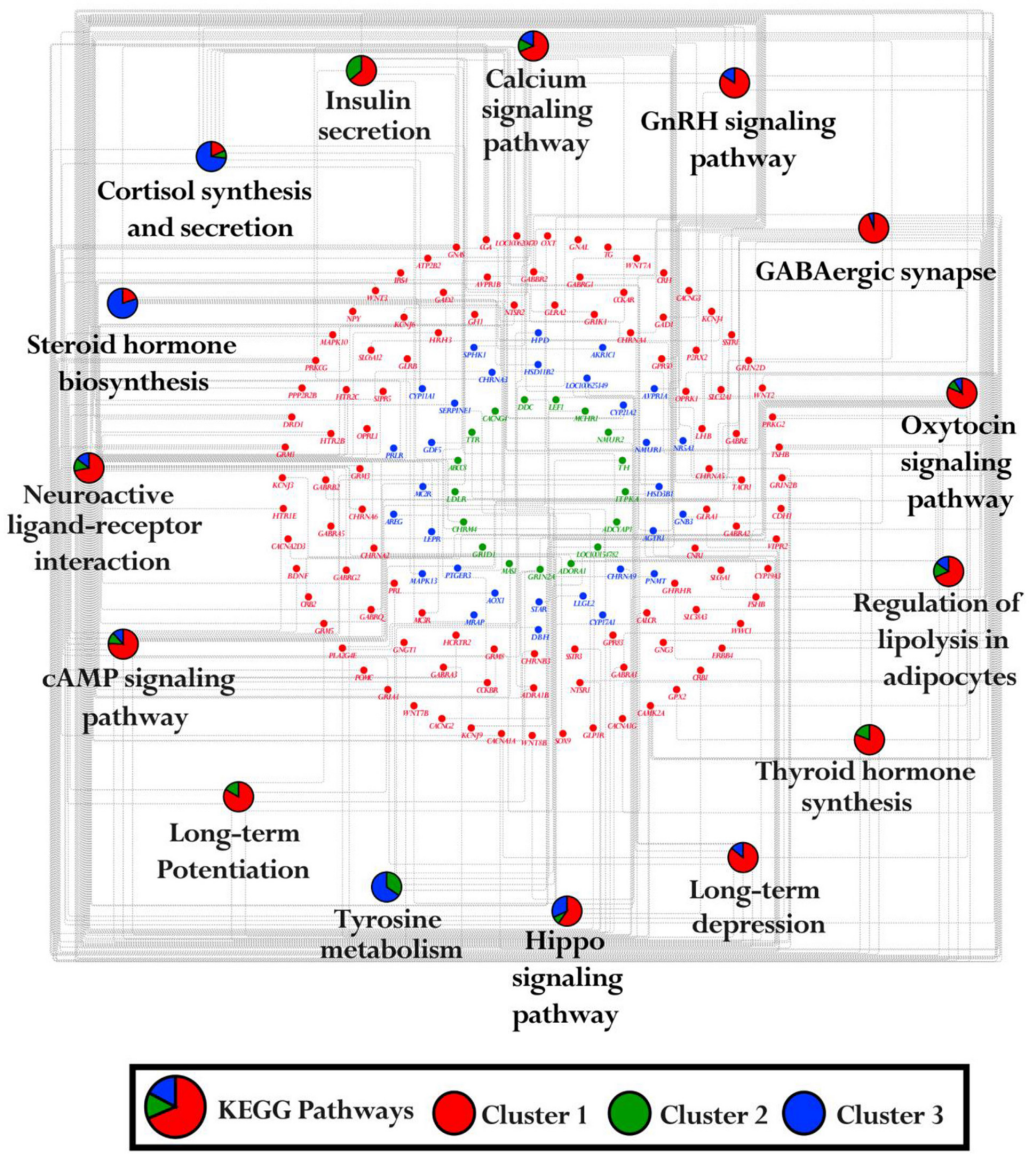
KEGG pathways enrichment network. The network illustrates the enrichment of the KEGG pathway for each cluster identified in the heatmap. The pie charts highlight gene count and their proportion in clusters C1, C2, and C3 across enriched pathways. The red ellipse indicated the genes associated with Cluster 1 (692 genes), the green ellipse indicated the genes in Cluster 2 (121 genes) and the blue ellipse indicated the genes in Cluster 3 (226 genes).

The KEGG pathway enrichment analysis was performed on genes from each cluster of the heatmap (C1: 692 genes, C2: 121 genes, and C3: 226 genes) using ClueGO (version 2.5.10) and Cluepedia (version 1.5.10) plugin in Cytoscape (version 3.10.2) environment. A total of 15 KEGG pathways were significantly enriched with a threshold of *p*-value < 0.05. These pathways include calcium signaling pathway, insulin secretion, cortisol synthesis and secretion, steroid hormone biosynthesis, neuroactive ligand-receptor interaction, cAMP signaling pathway, long-term potentiation, tyrosine metabolism, hippo signaling pathway, long-term depression, thyroid hormone synthesis, regulation of lipolysis in adipocytes, oxytocin signaling pathway, GABAergic synapse, and GnRH signaling pathway (Figure 3). Details on cluster proportion and gene counts within enriched pathways were provided in Supplementary Table S4.

Interestingly, the genes in cluster one (C1), which show higher expression levels in the amygdala, hippocampus, thalamus, and hypothalamus but lower expression in the pituitary and adrenal glands (Figure 2), are enriched in pathways such as oxytocin signaling, cAMP signaling, long-term potentiation, GABAergic synapse, long-term depression, Hippo signaling, and neuroactive ligand-receptor interaction. These pathways play a crucial role in regulating limbic system function. The genes from cluster two (C2) and cluster three (C3) that show higher expression in the PG and AG were enriched in cortisol synthesis and secretion, steroid hormone biosynthesis, and tyrosine metabolism. These pathways were involved in regulating the hormonal functions within the endocrine system (Figure 3).

### 3.3 Shared DEGs across multiple pairwise comparisons

**Figure 4:**
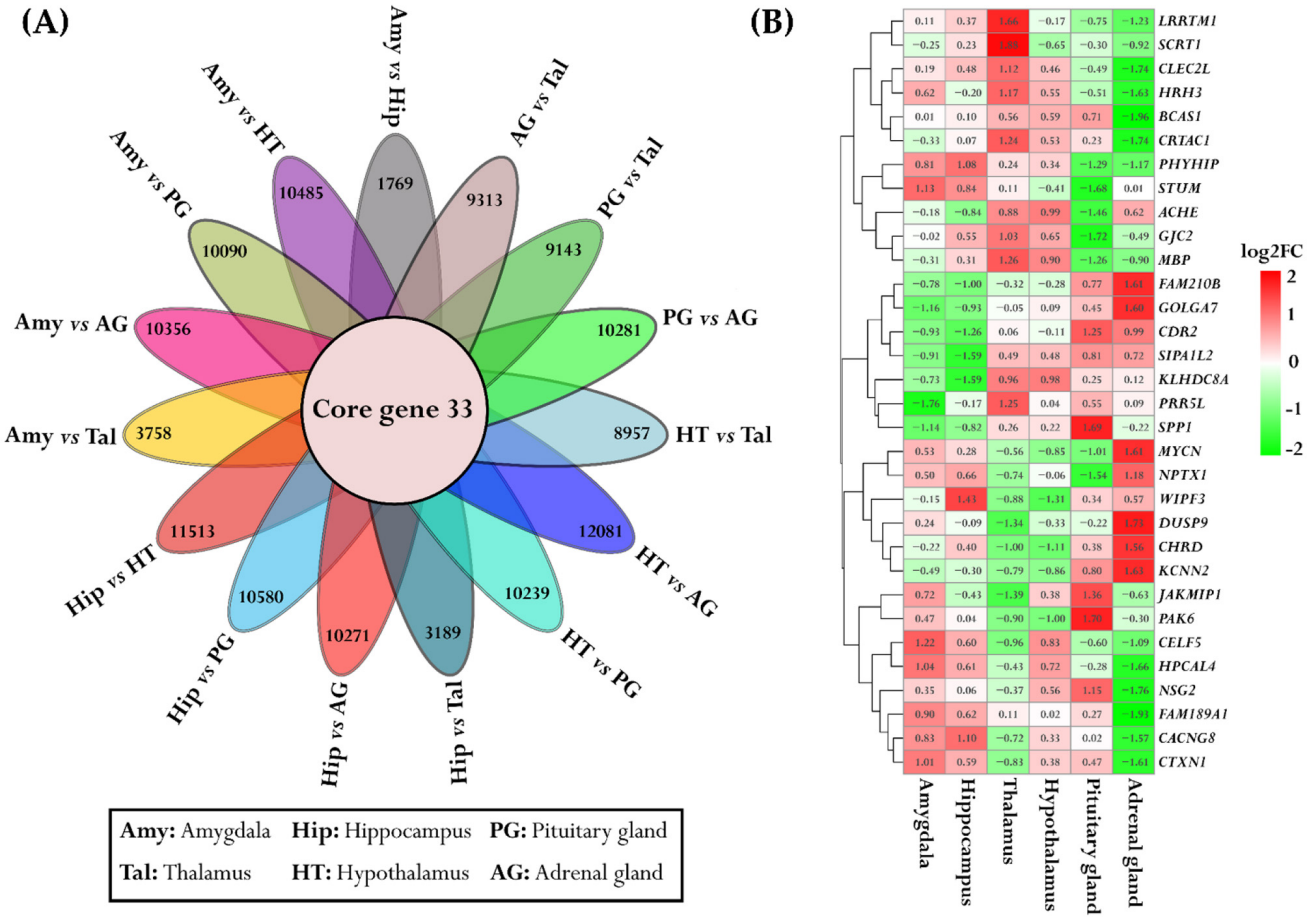
Overlapping genes and their expression across multiple tissues. **(A)** The flower plot depicts the core genes common across all pairwise tissue comparisons. Each petal of the flower represents differentially expressed genes between tissues, with the inner circle highlighting the core genes shared among all comparison groups. **(B)** The heatmap illustrates the expression patterns of these core genes across six different tissues. The color scheme is red for upregulation, green for downregulation, and white for no change.

The identification of core DEGs that overlapped across all 15 pairwise comparison groups revealed 33 genes that were consistently differentially expressed across all groups, as shown in Figure 4A. Additionally, the expression patterns of these 33 genes across all six tissues were visualized by a heatmap. In the amygdala and hippocampus, 3/33 genes include CUGBP Elav-Like Family Member 5 *(CELF5)*, Hippocalcin Like 4 *(HPCAL4)*, and Cortexin 1 *(CTXN1)* were genes that exhibit higher expression levels in the amygdala, while WASL interacting protein family member 3 *(WIPF3)* and Calcium Voltage-Gated Channel Auxiliary Subunit Gamma 8 *(CACNG8)* were genes that demonstrated higher expression in the hippocampus. Additionally, Phytanoyl-CoA Dioxygenase Domain-Containing Protein 1 *(PHYHIP)*, and Storkhead Box Homolog 1 *(STUM)* exhibit similar higher expression patterns in both the amygdala and hippocampus. Among the 33 common genes, 6 genes exhibited notably higher expression specifically in the thalamus, including Leucine Rich Repeat Transmembrane Neuronal 1 *(LRRTM1)*, Secretin Receptor Transmembrane Adaptor 1 *(SCRT1)*, C-type Lectin Domain Family 2 Member L *(CLEC2L)*, Histamine Receptor H3 *(HRH3)*, Cartilage Acidic Protein 1 *(CRTAC1)*, and Proline-Rich 5 Like *(PRR5L)*. Also, 4/33 genes including Acetylcholinesterase *(ACHE)*, Gap Junction Protein Gamma 2 *(GJC2)*, Myelin Basic Protein *(MBP)*, and Kelch Domain Containing 8A *(KLHDC8A)*, were genes that showed higher expression levels in both the thalamus and hypothalamus (Figure 4B).

In the adrenal gland and pituitary gland, 7/33 genes, encompassing Family with sequence similarity 210 member B *(FAM210B)*, Golgin A7 *(GOLGA7)*, Neuroblastoma MYC Oncogene *(MYCN)*, Neuronal Pentraxin 1 *(NPTX1)*, Dual Specificity Phosphatase 9 *(DUSP9)*, Chordin *(CHRD)*, and Potassium Calcium-Activated Channel Subfamily N Member 2 *(KCNN2)*, indicating higher expression levels in the adrenal gland, whereas in pituitary gland the following four genes showed higher expression: Secreted Phosphoprotein 2 *(SPP2)*, Janus Kinase and Microtubule-Interacting Protein 1 *(JAKMIP1)*, p21-Activated Kinase 6 *(PAK6)*, and Neuronal Synaptogyrin 2 *(NSG2)*. Only the Cerebellar Degeneration-Related Protein 2 *(CDR2)* gene indicated higher expression in both the adrenal gland and pituitary gland (Figure 4B).

### 3.4 Differential gene expression and functional enrichment in pairwise tissue group comparisons

**Figure 5:**
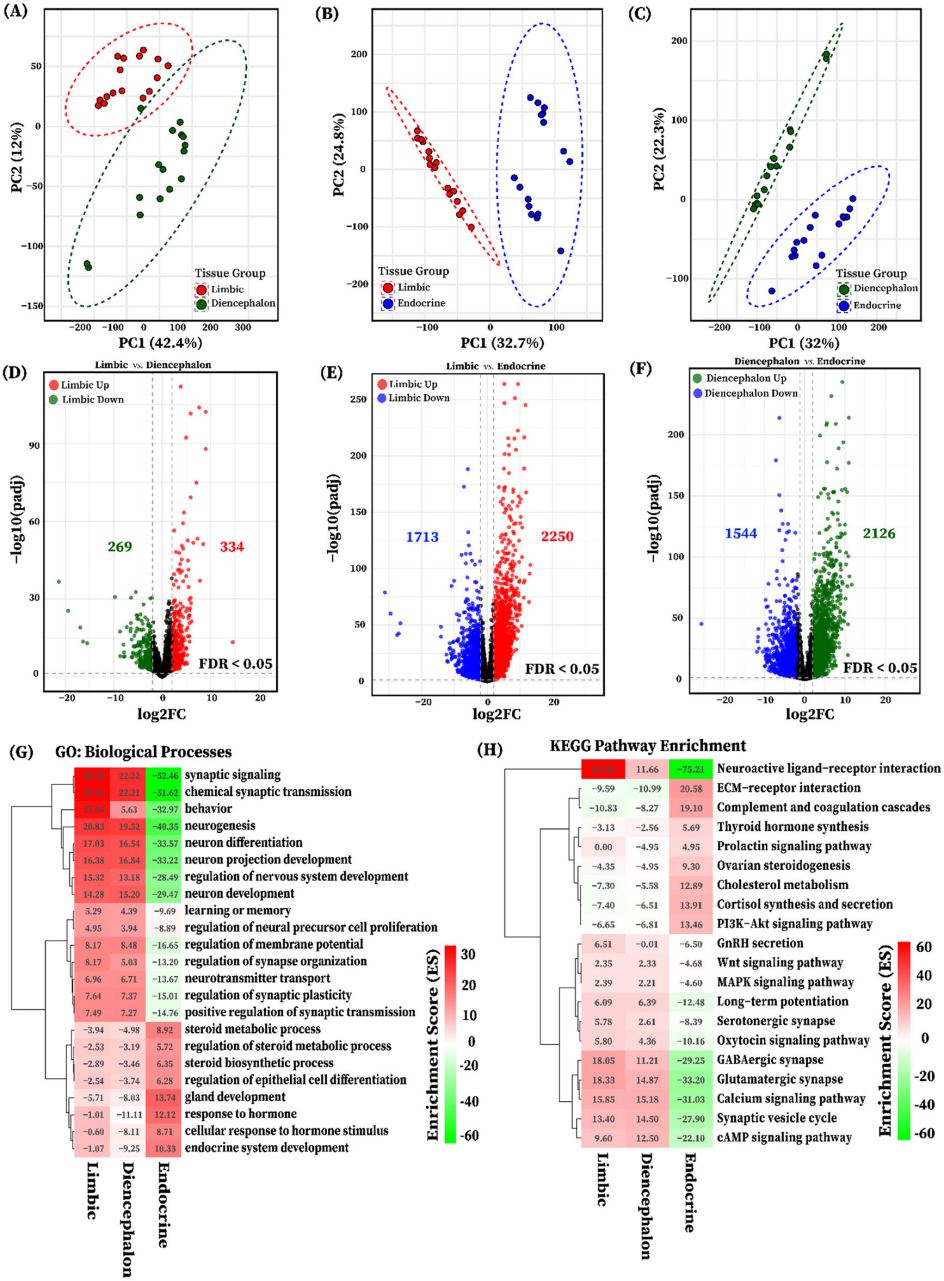
PCA and differential expression analysis of functional tissue groups. Principal component analysis (PCA) of variance-stabilized gene expression showing separation between tissue groups **(A)** Limbic and Diencephalon, **(B)** Limbic and Endocrine, **(C)** Diencephalon and Endocrine. Groups are color-coded: red (Limbic), dark green (Diencephalon), and blue (Endocrine). Volcano plots show differentially expressed genes (DEGs) for the pairwise tissue group comparisons **(D)** Limbic vs. Diencephalon (red: upregulated in Limbic; dark green: downregulated), **(E)** Limbic vs. Endocrine (red: upregulated in Limbic; blue: downregulated), and **(F)** Diencephalon vs. Endocrine (dark green: upregulated in Diencephalon; blue: downregulated). DEGs in each tissue group pairwise comparison are identified based on an FDR < 0.05 and |log_2_FC| > 2. Heatmaps illustrate the functional enrichment of differentially expressed genes (DEGs) identified from pairwise tissue group comparisons: **(G)** gene ontology (GO) biological processes and **(H)** KEGG pathways, both with FDR < 0.05. Red indicates positive enrichment scores representing processes and pathways enriched by genes upregulated in the limbic and diencephalon groups, while green indicates negative enrichment scores corresponding to genes upregulated in the Endocrine group.

For a more comprehensive transcriptome analysis of six pig tissues, we categorized them into three groups based on their functional and anatomical characteristics: the limbic group (Amy and Hip), the diencephalon group (Tal and HT), and the endocrine group (PG and AG). PCA based on variance-stabilized counts revealed distinct clustering patterns among tissue groups in each pairwise comparison (Figures 5A–C). Pairwise differential expression analyses were conducted between the three tissue groups: limbic vs. diencephalon, limbic vs. endocrine, and diencephalon vs. endocrine. Genes were considered significantly differentially expressed if they met the criteria of false discovery rate (FDR) < 0.05 and |log_2_ fold change| ≥ 2, as illustrated in Figures 5D–F. A total of 4,954 differentially expressed genes (DEGs) were identified across all group comparisons. The highest number of DEGs was observed in the limbic vs. endocrine comparison (3,963 transcripts, Figure 5E), followed by diencephalon vs. endocrine (3,670 transcripts, Figure 5F), and limbic vs. diencephalon (603 transcripts, Figure 5D). Summary statistics for all DEGs (log_2_FC, FDR, baseMean) are provided in Supplementary Table S5.

Furthermore, to elucidate the biological relevance of transcriptional differences among tissue groups, we performed gene ontology (GO) and KEGG pathway enrichment analyses using DEGs identified from pairwise comparisons. All significantly enriched terms and pathways were considered based on an FDR < 0.05, and results were visualized using enrichment scores, which capture the direction and magnitude of functional bias across tissue groups (Figures 5G, H). GO enrichment analysis of genes upregulated in limbic and diencephalon tissues compared to endocrine revealed strong enrichment for neurodevelopmental and neuronal signaling processes, including synaptic signaling, neurogenesis, axon guidance, and regulation of neurotransmitter secretion. These terms reflect the neural specialization of these brain regions. In contrast, genes upregulated in the Endocrine tissue were significantly enriched for biological processes such as hormone response, steroid metabolic process, gland development, and endocrine system development, reflecting the tissue's specialized hormonal and secretory functions, as shown in Figures 5G, H.

Consistently, KEGG pathway enrichment analysis revealed that the upregulated DEGs were associated with neuronal function-related pathways, including neuroactive ligand-receptor interaction, glutamatergic and GABAergic synapses, long-term potentiation, as well as calcium and cAMP signaling. These pathways are essential for synaptic transmission, neural plasticity, and intracellular signaling. Distinctly, DEGs upregulated in endocrine tissues were significantly enriched in pathways including ECM-receptor interaction, complement and coagulation cascade, cholesterol metabolism, PI3K-Akt signaling, and hormone biosynthesis and secretion (e.g., cortisol synthesis, ovarian steroidogenesis, and thyroid hormone synthesis). These enrichments suggest active structural remodeling, immune system involvement, and endocrine functional regulation (Figures 5G, H). Complete enrichment results are available in Supplementary Table S6.

### 3.5 Tissue group-specific co-expression modules and functional enrichment

**Figure 6:**
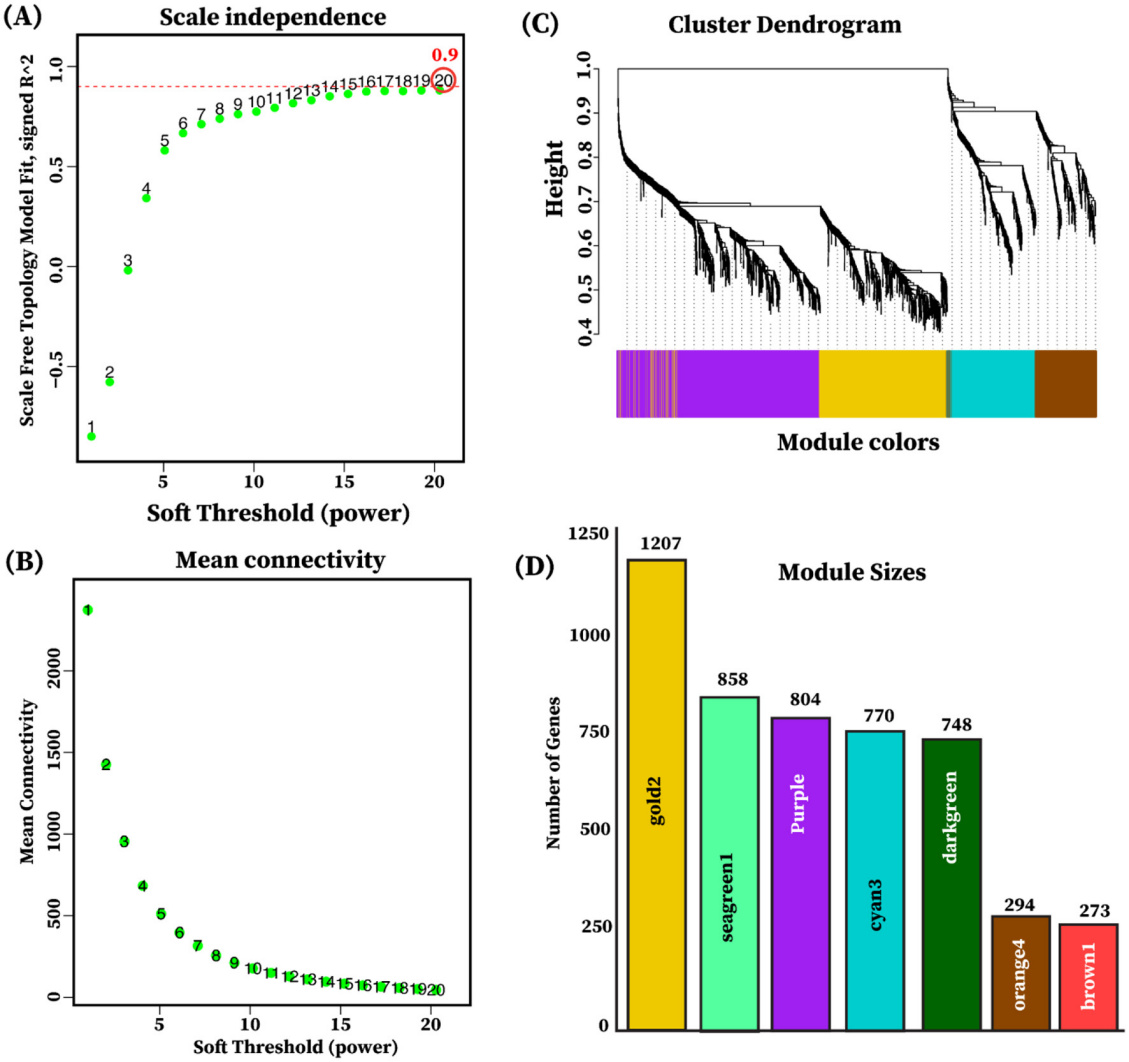
Selection of soft thresholding power and module detection. **(A)** Scale independence plot illustrating the determination of soft thresholding. The *y*-axis represents the scale-free topology, while the *x*-axis indicates the soft thresholding power. The red dotted line indicates the selected soft-thresholding power of (β = 20) where the scale-free topology fit index reached 0.9. **(B)** The mean connectivity plot shows the mean connectivity on the *y*-axis as a function of soft-thresholding power on the *x*-axis. **(C)** The cluster dendrogram of genes indicating the dissimilarity based on the topological overlap is utilized for module detection through dynamic tree cutting. Each color in the horizontal module colors bar below the dendrogram signifies a different module. **(D)** The bar plot illustrates each module, with the color of each bar indicating the module's color (*x*-axis) and its size representing the gene count (*y*-axis) within the module.

The weighted gene co-expression network analysis (WGCNA) was employed to explore the biological relationships and functional relevance of 4,954 differentially expressed genes (FDR < 0.05, |log_2_FC| ≥ 2) identified from pairwise comparisons among the three tissue groups: limbic, diencephalon, and endocrine. After evaluating the indices and mean connectivity across the powers ranging from 1 to 20, a soft thresholding value (β) of 20 was selected, corresponding to (*R*^2^ = 0.9), signifying a robust fit to the scale-free topology model and effectively balances scale independence and lower mean connectivity (Figures 6A, B). Furthermore, the gene co-expression modules were identified through hierarchical clustering by computing dissimilarity between genes derived from the transformed topological matrix (Figure 6C). A total of seven gene co-expression modules were identified as gold2, seagreen, purple, cyan3, darkgreen, orange4, and brown1, and the number of genes in each module ranged from 250 to 1,300 (Figure 6D, Supplementary Table S7).

**Figure 7:**
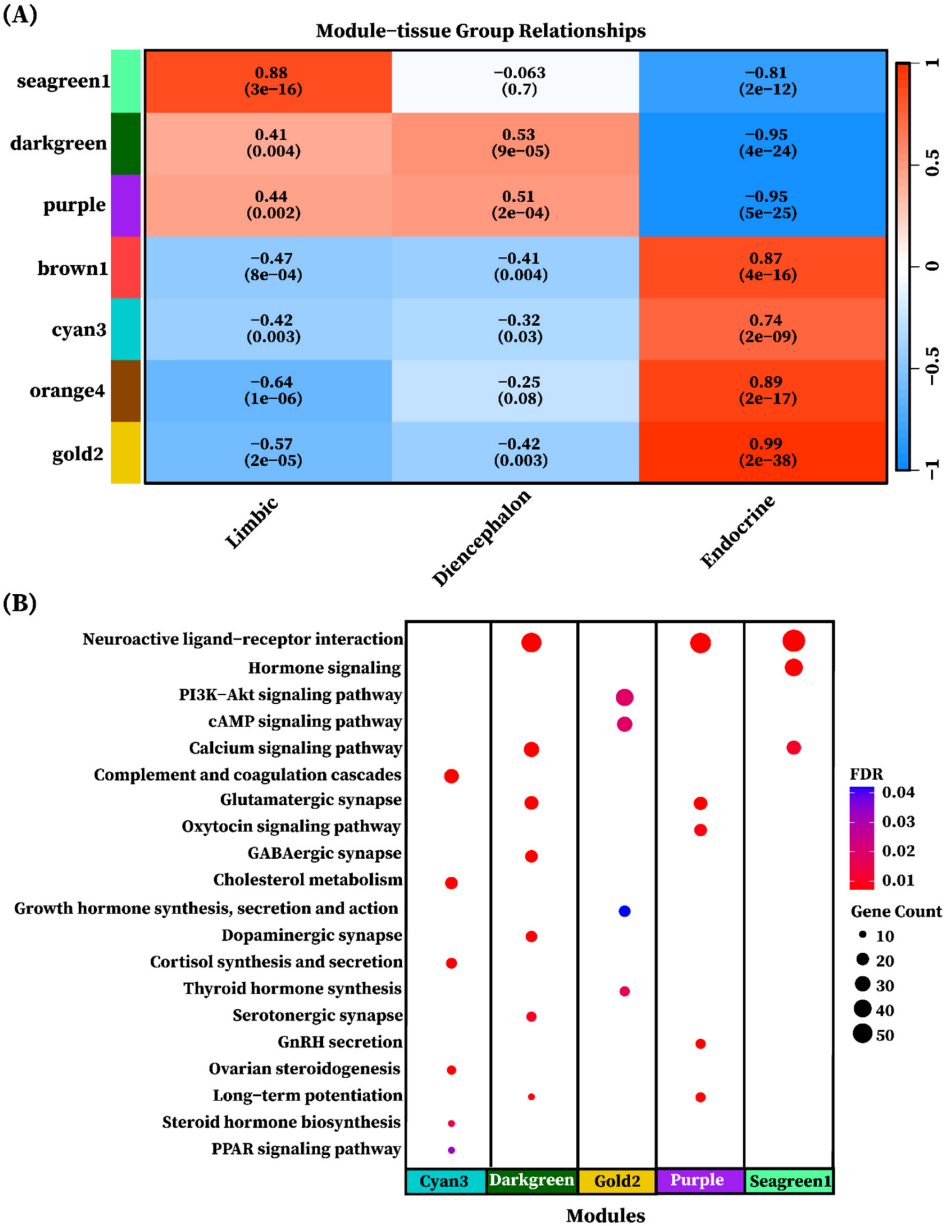
Heatmap of module eigengene-tissue group correlation. **(A)** The heatmap of eigengene adjacency shows the correlation between module eigengenes and tissue groups. The y-axis is labeled with color bars for modules (seagreen1, darkgreen, purple, brown1, cyan3, orange4, and gold2). Positive correlations are depicted in red shades, while negative correlations are indicated in blue shades. The color intensity corresponds to correlation strength, as indicated by the correlation bar. The significance of the correlation is represented by the *p*-values shown in brackets alongside with correlation value. **(B)** Each dot represents a KEGG pathway enriched in a different gene modules (cyan3, darkgreen, gold2, purple, and segreen1). The color of each dot represents the FDR-adjusted *p*-value and the size of the dot corresponds to the number of genes associated with each enriched pathway.

To explore tissue group–driven gene co-expression patterns, we correlated the eigengenes of the seven identified modules with each of the three tissue groups: limbic, diencephalon, and endocrine. Modules were considered group-specific if the correlation was statistically significant (*P* < 0.05). The analysis revealed that all seven modules displayed significant group-specific expression patterns, characterized by both positive and negative correlations. Positive correlations indicated upregulation in the corresponding tissue group, whereas negative correlations reflected relative downregulation. Among the identified modules, the seagreen1 module (858 genes) exhibited a strong positive correlation with the limbic group (*r* = 0.88, *P* = 3 × 10^−16^) and a strong negative correlation with the endocrine group (*r* = −0.81, *P* = 2 × 10^−12^, Figure 7A). It was enriched in Neuroactive ligand-receptor interaction (adjusted *p* = 1.84 × 10^−23^), Hormone signaling, and Calcium signaling pathway (Figure 7B). The darkgreen module (748 genes) showed moderate positive correlations with the limbic (*r* = 0.41, *P* < 0.01) and diencephalon (*r* = 0.53, *P* < 0.001) groups, and a strong negative correlation with the endocrine group (*r* = −0.95, *P* < 1 × 10^−24^, Figure 7A). It was enriched in Neuroactive ligand-receptor interaction (adjusted *p* = 5.01 × 10^−13^), Glutamatergic synapse, GABAergic synapse, Calcium signaling pathway, Dopaminergic synapse, Long-term potentiation, and Serotonergic synapse (Figure 7B). Purple module (804 genes) exhibited moderate positive correlations with the limbic (*r* = 0.44, *P* < 0.01) and diencephalon (*r* = 0.51, *P* < 0.001) groups, and a strong negative correlation with the endocrine group (*r* = −0.95, *P* < 1 × 10^−25^, Figure 7A). It was enriched in Neuroactive ligand-receptor interaction (adjusted *p* = 7.7 × 10^−12^), Glutamatergic synapse, GnRH secretion, Long-term potentiation, and Oxytocin signaling pathway (Figure 7B). Cyan3 module (770 genes) exhibited strong positive correlations with the endocrine group (*r* = 0.74, *P* = 2 × 10^−09^). It was enriched in Complement and coagulation cascades (adjusted *p* = 3.29 × 10^−10^), Cholesterol metabolism, Cortisol synthesis and secretion, Ovarian steroidogenesis, Steroid hormone biosynthesis, and PPAR signaling pathway. The gold2 module (1,207 genes) exhibited a very strong positive correlation with the endocrine group (*r* = 0.99, *P* = 2.41 × ^10 − 38^) and was enriched in Thyroid hormone synthesis (adjusted *p* < 0.01) PI3K-Akt signaling pathway, cAMP signaling pathway, and Growth hormone synthesis, secretion and action, as shown in Figures 7A, B. Notably, no significant enriched pathways were identified for the orange4 (294 genes) and brown1 (273 genes) modules despite their strong positive correlation with the endocrine group. The detailed enrichment results are provided in Supplementary Table S8.

### 3.6 Gene-based ASE analysis within tissues across individuals in the population

**Figure 8:**
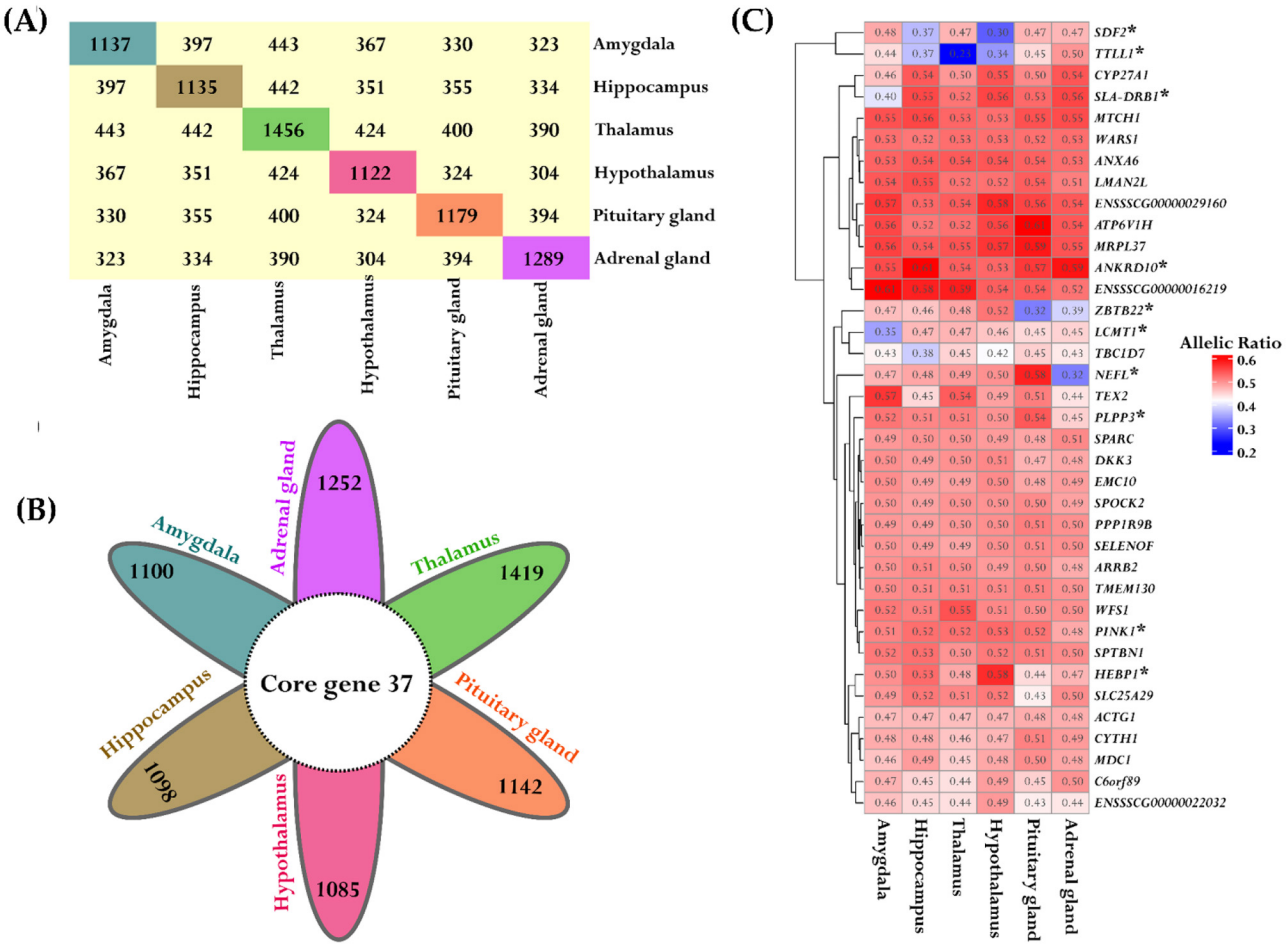
Consistent ASE patterns across different tissues. **(A)** The block plot displays the total number of ASE genes and overlapped genes between the tissues. The diagonal block in unique colors indicates the total number of ASE genes within each tissue. The off-diagonal yellow blocks represent the number of common genes between the tissues. **(B)** The flower plot represented the shared significant ASE genes across all tissues. Each petal corresponds to tissue-specific ASE genes and the inner circle depicts the overlapped genes. **(C)** The heatmap depicts the variation in allelic ratios of shared genes across all six tissues. The color bar indicates the strength of these differences, where red indicates higher allelic ratios and blue indicates lower allelic ratios. Significant genes are marked with a star for both tissue comparisons and overall tissue effects.

The data were analyzed using the “*ASE_detection()*” function for one-condition analysis from the ASEP package within the R environment, which conducts gene-level ASE analysis within the six tissues derived from the same population of eight animals. Significant ASE effects were identified in the following number of genes for each tissue: Amy (1,137), Hip (1,135), Tal (1,456), HT (1,122), PG (1,179), and AG (1,289), all at a significance level of *P* < 0.05. The detailed results are provided in Supplementary Table S9. We further examined the gene names that were shared across the different tissues. The distribution and quantity of shared ASE gene names between different tissues are summarized in Figure 8A. Additionally, we identified 37 genes that exhibited ASE and were shared across all examined tissues, as illustrated in Figure 8B. These genes were specifically selected for a detailed analysis, with the mean allelic ratio calculated for each tissue to reveal their expression profiles across diverse tissue environments (Figure 8C).

In addition, a variance analysis was conducted on these 37 genes with ASE to assess the overall impact of tissue type on allelic ratio variation. Pairwise comparisons between tissue types were also performed for each gene to examine differences in mean allelic ratios across tissues. Interestingly, 7 of the 37 genes showed a significant tissue-wide effect on allelic ratio (Supplementary Table S10). Furthermore, 10/37 genes in pairwise comparisons revealed significant differences in mean allelic ratio across tissues. The *PINK1* exhibits significant mean allelic ratio variation in all comparisons with the AG, including the comparison with the HT (*P* = 9.89e-05), Hip (*P* = 0.00041), Tal (*P* = 0.00045), PG (*P* = 0.0013), and Amy (*P* = 0.03). The mean allelic ratio differences for the Leucine Carboxyl Methyltransferase 1 (*LCMT1)* were determined in all comparisons with the Amy, including the comparison with the Tal (*P* = 0.0009), Hip (*P* = 0.0015), HT (*P* = 0.0043), AG (*P* = 0.0135), and PG (*P* = 0.022). The allelic ratio differences for the Zinc Finger And BTB Domain Containing 22 *(ZBTB22)* were observed in the Amy vs. PG (*P* < 0.0001), Hip vs. PG (*P* < 0.0001), Tal vs. PG (*P* < 0.0001), HT vs. PG (*P* < 0.0001), and HT vs. AG (*P* = 0.0003). Also, significant variations in the mean allelic ratio were observed for the *PPL3* gene between the AG vs. PG (*P* = 0.001) and AG vs. Amy (*P* = 0.02). The mean allelic ratio differences for the *HEBP1* were observed only in the HT vs. PG comparison (*P* = 0.013). Interestingly, 7/10 genes, including *PINK1, TTLL1, SLA-DRB1, HEBP1, ANKRD10, LCMT1*, and *SDF2*, exhibited ASE and were also recognized as eQTLs in brain tissue according to data from the PigGTEx portal within the FarmGTEx database, as outlined in Supplementary Table S10.

**Figure 9:**
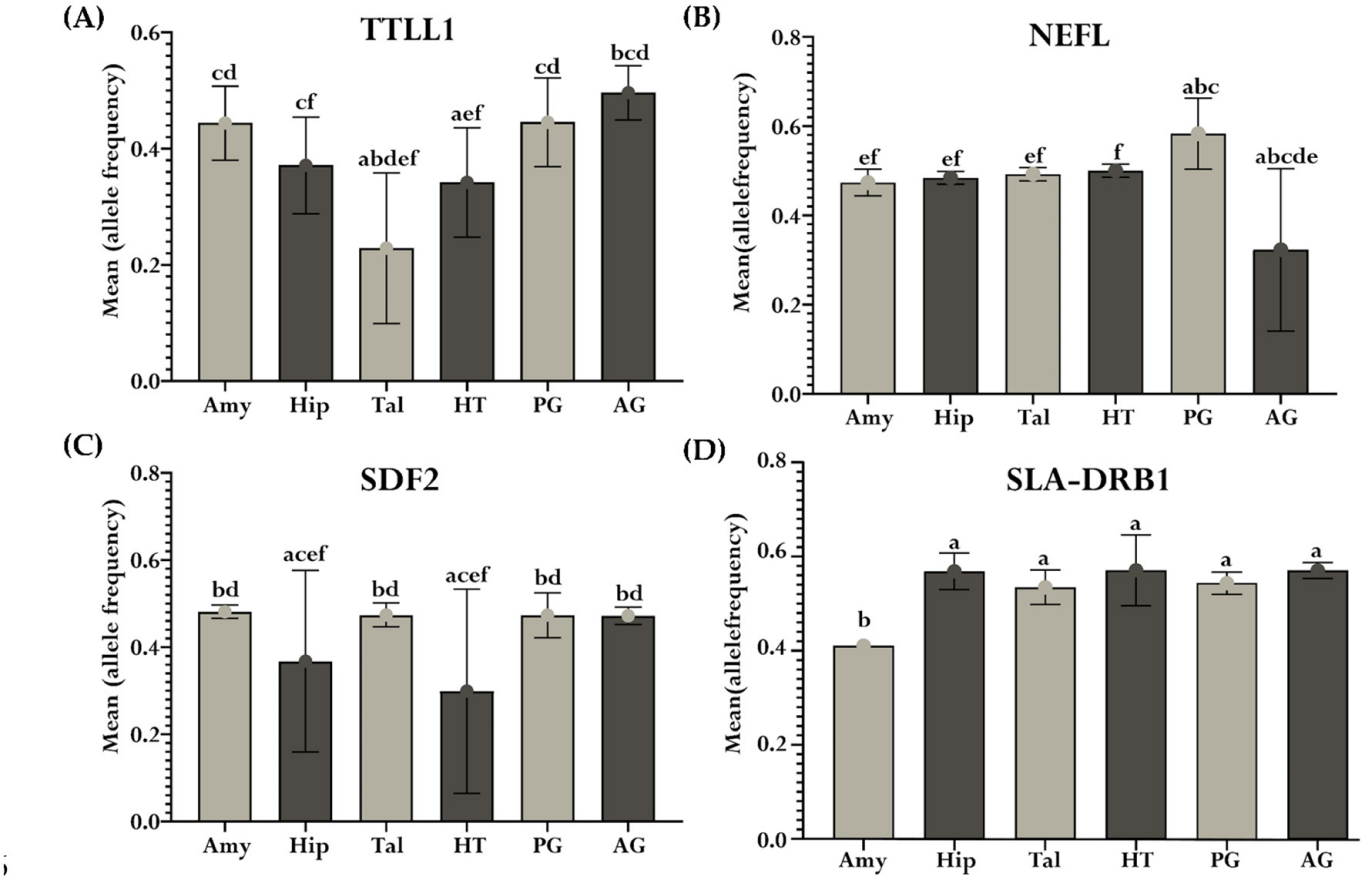
Mean allelic ratio variability across tissues for significant genes. Each bar plot (a–d) represents the significant mean allelic ratio differences of genes across the Amy, Hip, Tal, HT, PG, and AG. Subplot **(A)** depicts *TTLL1*, **(B)** *NEFL*, **(C)** *SDF2*, and **(D)** *SLA-DRB1*. Differences in the mean allelic ratio between tissues were represented by unique letters (“a”–“f”), where shared letters indicated non-significant differences from each other at *P* < 0.05. The error bars represent the standard deviation of the mean allelic ratio within each tissue.

Additionally, *TTL1, NEFL, SDF2*, and *SLA-DRB1* demonstrated notable differences in mean allelic ratio across analyzed tissues. *TTL1*, showed pronounced variations in comparisons such as Tal vs. Amy (*P* < 0.0001), Tal vs. Hip (*P* < 0.0001), Tal vs. HT (*P* = 0.0028), Tal vs. PG (*P* < 0.0001), Tal vs. AG (*P* < 0.0001), Amy vs. HT (*P* = 0.01), Hip vs. AG (*P* = 0.0007), HT vs. PG (*P* = 0.0091), and HT vs. AG (*P* < 0.0001, Figure 9A). The *NEFL* demonstrates the mean allelic ratio variation between Amy vs. PG (*P* = 0.0043), Amy vs. AG (*P* < 0.0001*)*, Hip vs. PG (*P* = 0.01), and Hip vs. AG (*P* < 0.0001), Tal vs. PG (*P* = 0.03), Tal vs. AG (*P* < 0.0001), HT vs. AG (*P* < 0.0001), and PG vs. AG (*P* < 0.0001, Figure 9B). The Stromal Cell Derived Factor 2 *(SDF2)* gene demonstrates notable variations in mean allelic ratio across various brain regions and glands. Significant differences were observed in comparisons between the Amy and both the Hip (*P* = 0.0035) and HT (*P* < 0.0001). Similarly, the significant differences were determined in the Hip vs. Tal (*P* = 0.0084), Hip vs. PG (*P* = 0.009), Hip vs. AG (*P* = 0.01), Tal vs. HT (*P* < 0.0001), HT vs. PG (*P* < 0.0001), and HT vs. AG (*P* < 0.0001, Figure 9C). The Swine Leukocyte Antigen Class II, DR Beta 1 (*SLA-DRB1)* gene exhibits significant mean allelic ratio variation in all comparisons with the Amy, including the comparison with the Hip (*P* < 0.0001), Tal (*P* = 0.0014), HT (*P* < 0.0001) PG (*P* = 0.0004), and AG (*P* < 0.0001, Figure 9D).

### 3.7 Tissue-specific ASE gene overlaps with brain, hypothalamus, and pituitary gland eQTLs from the PigGTEx database

**Figure 10:**
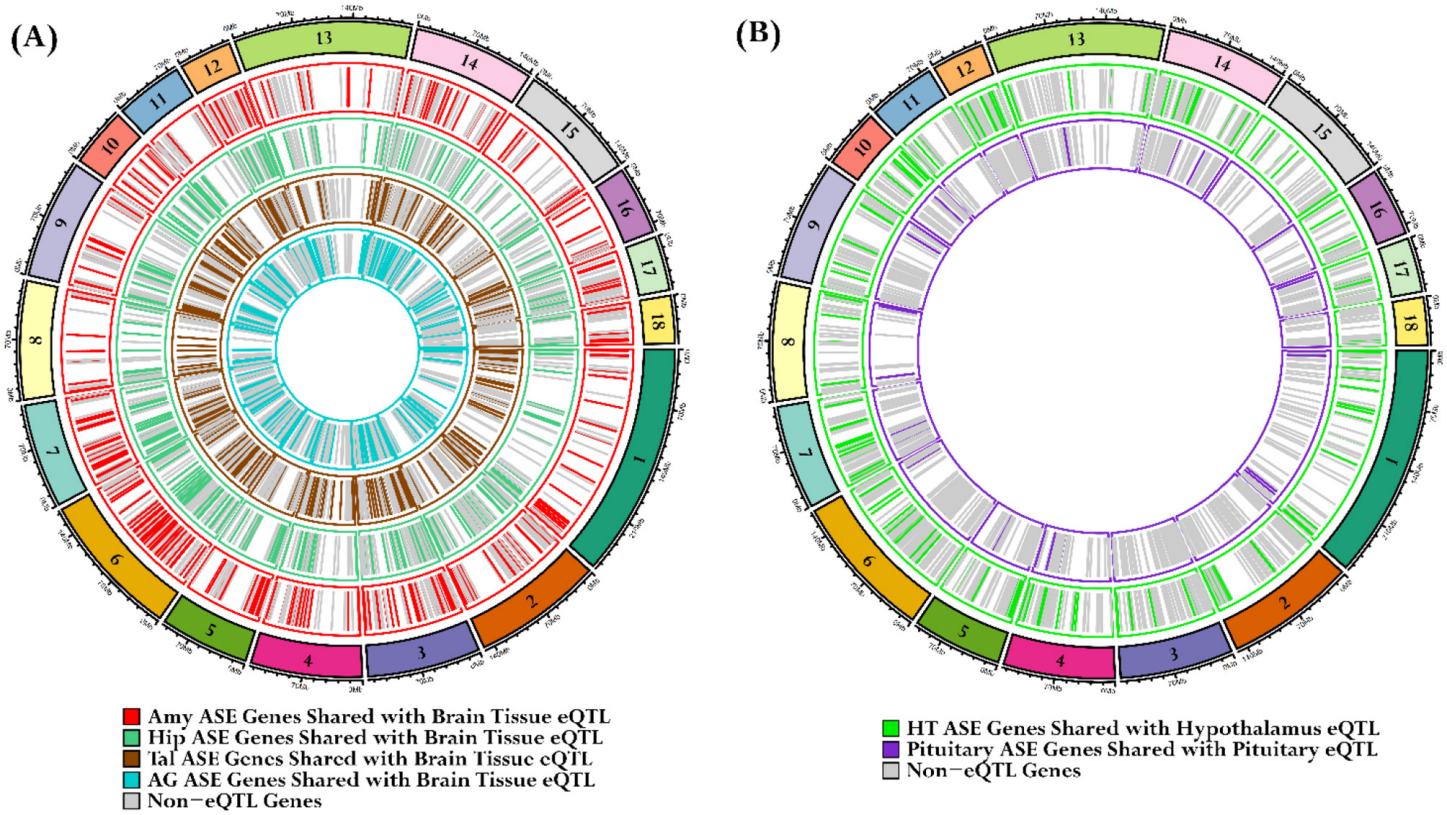
Identification of overlaps between tissue-specific ASE genes and PigGTEx database eQTLs. **(A)** The first circos plot illustrates the overlap of tissue-specific ASE genes with eQTLs from pig brain tissues in the PigGTEx database. Each of the four concentric layers represents a distinct tissue: red lines for the Amy, sea-green lines for the Hip, brown lines for the Tal, and cyan lines for the AG. **(B)** The subsequent circos plot focuses on the HT and PG. The green lines represent HTASE genes that overlap with hypothalamus eQTLs, while the purple lines denote PG ASE genes that overlap with pituitary gland eQTLs. The gray lines signify ASE genes not classified as eQTLs.

Our tissue-specific ASE analysis revealed that over a thousand genes exhibit allele-specific expression in brain tissues and endocrine glands, with a significance threshold of *P* < 0.05. We employed a gene-matching strategy to demonstrate that ASE genes from the Amy, Hip, Tal, and AG overlapped with brain tissue eQTLs data, from the PigGTEx portal, filtered at an FDR < 0.05. In Amy, 1,137 genes exhibited significant ASE, with 497 (43.7%) genes overlapping with brain eQTLs. The Hip had 1,135 genes with significant ASE, of which 492 (43.3%) were common with brain eQTLs. In the Tal, 1,456 genes exhibited significant ASE, with 619 (42.5%) genes shared with brain eQTLs. The AG had 1,289 genes with significant ASE, of which 544 (42.3%) were common with brain eQTLs, as shown in Figure 10A. In the HT, 1,122 genes with significant ASE, of which 213 (18.9%) were common with hypothalamus tissue eQTLs from the PigGTEx portal, were filtered at an FDR < 0.05. Similarly, the PG had 1,179 genes with significant ASE, of which 49 (4.1%) genes shared with pituitary gland eQTLs from the PigGTEx portal, filtered at an FDR < 0.05 (Figure 10B); detailed results are outlined in Supplementary Table S9.

## 4 Discussion

This study highlights the variation in genetic regulation between brain and endocrine tissues, emphasizing the complex interplay of genetic and regulatory mechanisms underlying stress adaptation and endocrine function. While previous studies on ASE in livestock have focused on tissues like the liver and muscle (Yang et al., 2016; Khansefid et al., 2018; Guillocheau et al., 2019; de Souza et al., 2020; Liu et al., 2020), our study extends these findings to limbic and endocrine tissues, revealing distinct ASE patterns indicative of tissue-specific regulation. Differential gene expression profiles across these tissues were identified, including co-expression network analysis.

Hierarchical clustering of differentially expressed genes across six tissues showed higher expression in limbic tissues compared to endocrine glands, with significant enrichment in pathways such as oxytocin signaling, GABAergic synapse, long-term depression (LTD), and long-term potentiation (LTP). Previous research has consistently shown that oxytocin signaling plays a critical role in modulating the limbic forebrain network, influencing stress responses, emotional behavior, and social interactions (Burkett et al., 2016; Bakos et al., 2018; Ferrer-Pérez et al., 2020; Triana-Del Rio et al., 2022). Additional studies have found that oxytocin alters synaptic plasticity through its effects on LTP and LTD, and promotes LTD in the amygdala via Gαq/11-coupled PLC and EGFR pathways which are essential for synaptic plasticity in the hippocampus (Lin et al., 2012; Gur et al., 2014), and modulates GABAergic activity in the mPFC, aiding in threat extinction in both humans and rodents (Sabihi et al., 2017; Eckstein et al., 2019). Our findings support these studies by demonstrating that oxytocin signaling and its related pathways are crucial for stress resilience and emotional health. Additionally, prior research has established cortisol's key role in stress response and physiological balance (McEwen, 1998; Smith and Vale, 2006; Knezevic et al., 2023). Consistent with these, we identified genes highly expressed in the pituitary and adrenal glands that are enriched in pathways for cortisol synthesis and steroid hormone biosynthesis, highlighting their importance in stress resilience.

The identification of a core set of 33 genes differentially expressed across all tissue comparisons, emphasizes their involvement in neural activity and stress regulation. In the amygdala, both *CELF5* and *HPCAL4* exhibited notably high expression. *CELF5*, a member of a gene family involved in RNA regulation and synaptic plasticity, is likely to contribute to emotional regulation (Bryant and Yazdani, 2016; Parra and Johnston, 2022; Peng et al., 2024). Recent single-cell transcriptomic analysis of the mouse brain supports this, showing that *CELF1* is broadly expressed, *CELF2* is enriched in neurons, and *CELF3–6* are variably present in neurons and neuroblast cells (La Manno et al., 2021). Likewise, *HPCAL4*, a key calcium-binding protein involved in neurotransmitter release and LTP critical for learning and memory (Burgoyne, 2007; Alvaro et al., 2020), aligns with its observed higher expression in this region, supporting its role in neural function. In the hippocampus, high expression levels of *WIPF3* and *CACNG8* were observed. *WIPF3*, which complexes with N-WASP, plays a critical role in regulating the actin cytoskeleton (Juszczak and Stankiewicz, 2018), a process essential for synaptic function, learning, and memory (Lamprecht, 2011, 2014, 2021), increased *WIPF3* expression may be enhanced WIPF3-N-WASP complex activity, potentially influencing synaptic plasticity and memory formation. Similarly, bioinformatics and functional studies have shown that members of the *CACNG* protein family (*CACNG1–CACNG8*) are co-expressed in adult brains to regulate Ca^2+^ channel activity (Burgess et al., 2001; Guan et al., 2016), suggesting that *CACNG8* may also contribute to synaptic transmission, plasticity, and the adaptation of neural networks. Our analysis revealed high *LRRTM1* expression in the thalamus, highlighting its significant role in neural connectivity and thalamic function. *LRRTM1* is essential for synaptic adhesion and signaling, which are critical for effective sensory processing, a finding consistent with previous studies showing its high abundance in the thalamus, particularly in the mediodorsal nucleus across multiple species (Laurén et al., 2003; Francks et al., 2007; Sjostedt et al., 2020). Furthermore, knockout studies have demonstrated that deletion of *LRRTM1* results in notable alterations in synapse morphology, impairments in novel object recognition and social interaction (Takashima et al., 2011), and visual behavior abnormalities due to disrupted retinothalamic connections (Monavarfeshani et al., 2018). Collectively, these results emphasize LRRTM1's critical role in thalamic functionality and its broader implications in neural processes. Previous studies have shown that severe inflammatory conditions, such as sepsis, significantly reduce *ACHE* activity in the hypothalamus, evidenced by notable decreases 5 days post-cecal ligation and puncture in rats, indicating cholinergic disruption (Santos-Junior et al., 2018). Similarly, low-dose LPS administration in mice leads to neuroinflammation and diminished cortical *ACHE* activity, emphasizing the vulnerability of the cholinergic system (Lykhmus et al., 2016). In contrast, our observation of elevated baseline *ACHE* expression in the hypothalamus suggests a critical role in maintaining cholinergic stability and potentially managing inflammatory disturbances.

The MKK6/p38 pathway stimulates *PAK6*, a key regulator of cellular stress responses (Kaur et al., 2005). Its elevated expression in the pituitary suggests a critical role in stress response mechanisms and endocrine regulation. Similarly, increased *SPP1* expression in the pituitary may modulate function via activation of the MAPK signaling pathway, known for its roles in inflammation and neuroprotection (Meller et al., 2005). In the adrenal gland, elevated levels of *DUSP9*, a key modulator of MAPK signaling linked to cellular stress and insulin resistance, suggest a role in regulating stress-related signaling and metabolic processes. Furthermore, our observation of increased *KCNN2* expression aligns with studies showing that overexpression of the *SK2* channel reduces stress-induced corticosterone secretion (Morel et al., 2019; Zhang et al., 2019), while SK2 infusion leads to lower corticosterone levels (Mitra et al., 2009). Together, these results offer a comprehensive view of gene expression across tissues and highlight the coordinated roles these genes play in neural activity, synaptic adaptation, and stress regulation.

Our comparative DEG analysis between the limbic diencephalon and endocrine groups highlights extensive transcriptional differentiation, indicative of their respective roles in neural circuitry and endocrine signaling. Previous studies have shown that learning and memory rely on synaptic plasticity mediated by activity-dependent calcium influx through NMDA and AMPA receptors (Hunt and Castillo, 2012; Kennedy, 2016; Wang and Peng, 2016). Consistent with this, our KEGG pathway enrichment analysis showed that DEGs upregulated in the limbic and diencephalon groups were significantly enriched in pathways involved in synaptic signaling and plasticity, including glutamatergic synapse, calcium signaling, and long-term potentiation, highlighting the molecular specialization of these brain regions for cognitive and neural processing functions. Within the neuroendocrine axis, the intermediate transcriptional state of the diencephalon group supports its integrative role in neurohormonal regulation. As a center of the limbic system, the hypothalamus links the endocrine and nervous systems to maintain homeostasis and regulate stress, immune responses, autonomic functions, and hormone-driven processes such as growth, fluid balance, and lactation (Kullmann et al., 2014; Soto-Tinoco et al., 2016). In line with these functions, our analysis revealed enrichment of neuroactive ligand–receptor interaction, calcium signaling, and synaptic plasticity pathways in the diencephalon group. These findings reflect the hypothalamus's ability to integrate signals from multiple brain regions and convert them into hormonal outputs that guide pituitary regulation of thyroid, adrenal, and reproductive organs. Furthermore, the observed enrichment of ECM–receptor interaction pathways in our endocrine tissues aligns with evidence from a rodent study showing that extracellular matrix proteins and integrin signaling enhance ACTH-induced cortisol secretion in adrenocortical cells (Otis et al., 2007). This suggests that in pigs, ECM remodeling and integrin-mediated signaling may similarly support adrenal responsiveness to ACTH stimulation, facilitating rapid glucocorticoid release during stress. Also, our analysis showed increased PI3K–Akt signaling and cholesterol metabolism in endocrine tissues, key pathways for steroid hormone production and stress response. Similarly, a sheep study found that PI3K–Akt and MEK/ERK pathways regulate ACTH-driven cortisol release and eNOS activity, highlighting their importance in adapting to stress (Newby et al., 2015). Overall, these enrichment results demonstrate the pivotal role of the hypothalamic–pituitary–adrenal (HPA) axis in cortisol regulation and stress resilience, reflecting the specialized transcriptional profiles that support neuroendocrine function in pigs.

Our weighted gene co-expression network analysis demonstrates a strong tissue-driven organization of co-expression networks within the limbic-diencephalon-endocrine axis. The significant enrichment of synaptic signaling pathways (Neuroactive ligand-receptor interactions, Glutamatergic/GABAergic synapses, Calcium signaling) within modules positively correlated with limbic/diencephalon groups (seagreen1, darkgreen, purple) robustly supports previous findings that identify these pathways as essential for neural communication and plasticity in these brain regions (Lein et al., 2007; Südhof, 2018). Also, the strong negative correlations observed with the endocrine group suggest a transcriptional trade-off that highlights the distinct functional roles of neural vs. endocrine pathways (Miller et al., 2014; Hodge et al., 2019). The significant enrichment of cortisol synthesis and secretion within the endocrine-correlated cyan3 module (*r* = 0.74, *P* = 2 × 10^−9^) is particularly notable, as cortisol represents the primary glucocorticoid mediating vertebrate stress adaptation (Dedovic et al., 2009). The co-enrichment of cholesterol metabolism (a cortisol precursor) (Gomez-Sanchez and Gomez-Sanchez, 2024) and PPAR signaling (involved in metabolic stress regulation) (Camps et al., 2012), within this module, suggests coordinated transcriptional regulation of integrated stress response pathways. Similarly, the gold2 module (*r* = 0.99, *P* = 2.41 × 10^−38^) is enriched for cAMP signaling a key second messenger involved in stress hormone secretion (Kutsukake et al., 2023) as well as pathways related to growth hormone and thyroid hormone synthesis, all of which contribute to metabolic stress adaptation (Tavares et al., 2023). These pathways offer key molecular targets to improve stress resilience in pigs, with direct benefits for animal welfare and production efficiency under challenging conditions.

Among the 37 genes showing ASE across all tissues, seven demonstrated a significant overall tissue effect, while 10 showed tissue-specific differences. The remaining 27 genes exhibited consistent ASE due to general allelic imbalances or uniform regulatory mechanisms across tissues. Notably, the differential *PINK1* allelic ratios between the adrenal gland and other tissues (HT, Hip, Tal, PG, Amy) suggest tissue-specific genetic regulation. This is supported by strong correlations between *PINK1* expression and stress hormones (corticosterone: *r* = 0.879; adrenaline: *r* = 0.881), as well as evidence that *PINK1*-deficient mice are more vulnerable to corticosterone-induced depression (Agnihotri et al., 2019). Furthermore, *PINK1* is recognized as an eQTL in pig brain tissue (Teng et al., 2024), further emphasizing its involvement in stress and hormonal responses. Similarly, *LCMT1* showed significant mean allelic ratio differences in the amygdala compared to other tissues, suggesting its role in neuroprotection and stress response. As an eQTL in pig brain tissue (Teng et al., 2024), *LCMT1* is also implicated in neurodegenerative diseases such as Alzheimer's (Nicholls et al., 2016) and manganese-related neurotoxicity (Xu et al., 2021; Zhang et al., 2023), highlighting its importance for brain function and neuroprotection (Sontag et al., 2008; Gnanaprakash et al., 2021). In our study, *LCMT1* exhibited a mean allelic ratio variation in the amygdala (65% vs. 35%), which may influence its role in neuroprotection and stress resilience, especially in the amygdala, a key region for emotional regulation.

The allelic imbalance of *ZBTB22* in the pituitary (68% vs. 32%) and adrenal glands (61% vs. 39%) suggests a potential impact on endocrine function and stress pathways, in line with its known roles in cellular metabolism and oxidative stress (Guo et al., 2023; Liu et al., 2023). *TTL1* exhibited significant mean allelic ratio variations across multiple brain regions with *P* < 0.01. Its critical role in neural development is highlighted by *TTL1*-null mice, which exhibit severe developmental defects and early post-natal death due to disorganized neuronal networks (Erck et al., 2005; Fukushima et al., 2009). Additionally, *TTL1* is identified as an eQTL in pig brain tissue (Teng et al., 2024), suggesting that its allelic variation may influence gene regulation across regions, consistent with our findings. Furthermore, the mean allelic ratio variations of *NEFL* in the adrenal and pituitary glands align with previous findings showing elevated levels of neurofilaments (*NEFL, NEFM, NEFH*) in chronically stressed mice and the cerebrospinal fluid of trauma-exposed individuals (Zetterberg et al., 2013), suggesting a role for *NEFL* in regulating the hypothalamic-pituitary-adrenal (HPA) axis. Previous studies have shown that ER stress in the hypothalamus disrupts energy balance by affecting leptin signaling, leading to sympathetic nervous system inhibition, reduced brown adipose tissue (BAT) thermogenesis, and weight gain (Contreras et al., 2014; González-García et al., 2017; Cakir and Nillni, 2019). Similarly, in the hippocampus, ER stress is associated with impaired insulin signaling and increased inflammation, particularly with high-fat diets (Nakandakari et al., 2019). The ASE of *SDF2*, observed at 70:30 in the hypothalamus and 63:37 in the hippocampus in our study, along with its identification as a pig brain eQTL (Teng et al., 2024), suggests it may help mitigate ER stress, preserve leptin signaling, and reduce inflammation and insulin resistance. Finally, the significant allelic ratio variation of *SLA-DRB1* in the amygdala, combined with its known importance in regulating pro-inflammatory cytokines (e.g., IL-1β, IL-6, TNF-α) and microglial activation (Harrison et al., 2009; Inagaki et al., 2012; Hu et al., 2022; Nazir et al., 2022), supports its involvement in immune regulation and stress-related mood disorders. *SLA-DRB1* is also recognized as an eQTL in pig brain tissue (Teng et al., 2024), further emphasizing its potential impact on gene regulation in stress responses. The overarching goal of incorporating ASE alongside DEG and WGCNA is to gain a multi-layered understanding of gene regulation in pig stress biology. While DEG and WGCNA highlight transcriptional changes and gene co-regulation, ASE uncovers cis-regulatory variants that may drive tissue-specific expression. Together, these approaches help identify robust candidate genes and pathways for improving animal welfare and breeding strategies.

## 5 Conclusions

Our findings underscore the molecular basis of stress regulation in pigs by highlighting gene expression and allele-specific activity across both individual tissues and functional groups, including the limbic, diencephalon, and endocrine regions. Through a multifaceted analysis of gene expression, co-expression, and ASE, we identified key genes and regulatory modules involved in stress processing, growth, and hormonal signaling—insights that have practical implications for improving animal welfare. Specifically, critical pathways such as MAPK, JAK-STAT, and NF-κB were found to play central roles in stress and inflammatory responses. Genes including *CELF5, PINK1*, and *LRRTM1* exhibited tissue-specific roles related to synaptic plasticity, neuroprotection, and hormonal regulation.

In addition, the discovery of significant allelic ratio variations across tissues highlights underlying genetic factors that may influence stress resilience in a tissue-specific manner. Notably, genes such as *LCMT1, TTL1, SLA-DRB1*, and *SDF2* not only showed ASE but are also classified as eQTLs in the PigGTEx portal (FarmGTEx database), suggesting their functional regulatory relevance. Identifying stress-responsive pathways and cis-regulatory variation offers valuable opportunities to breed more resilient animals, enhance environmental enrichment strategies, and tailor dietary interventions. These approaches, rooted in molecular insights, can help reduce chronic stress, improve growth and reproductive outcomes, and ultimately support more sustainable and ethical pig farming practices.

## Data Availability

The datasets presented in this study can be found in online repositories. The names of the repository/repositories and accession number(s) can be found in the article/Supplementary material. The RNA sequencing data were deposited in the ArrayExpress database under the provided accession: E-MTAB-14452.
